# Magnolia essential oil: a preliminary exploration of chemical composition and its antimicrobial and antioxidant potential

**DOI:** 10.3389/fmicb.2025.1509796

**Published:** 2025-04-10

**Authors:** Yingjie Liu, Ningling Han, Fanxin Meng

**Affiliations:** ^1^College of Pharmacy and Food Science, Zhuhai College of Science and Technology, Zhuhai, China; ^2^College of Life Science, Jilin University, Changchun, China

**Keywords:** magnoliae flos, essential oil, GC-MS, chemical composition, antibacterial, antioxidant

## Abstract

In this study, the chemical composition of Magnolia essential oil (MEO) was analyzed using gas chromatography-mass spectrometry (GC-MS). The results indicated that terpenoids were the primary constituents, with the main components being 1,8-cineole (44.87%), (+)-citronellal (6.93%), and linalool (29.1%). The antibacterial activity of MEO against four target bacteria was confirmed through inhibition zone assays, minimum inhibitory concentration (MIC), and minimum bactericidal concentration (MBC) tests. The bacterial growth curve demonstrated that MEO significantly inhibited bacterial growth and effectively delayed the logarithmic growth phase. Mechanistic studies suggested that MEO primarily acts in the initial stages of bacterial growth by disrupting the bacterial cell membrane, leading to substantial leakage of intracellular materials, impairing metabolic activities, inducing lipid peroxidation, and enhancing oxidative stress, thereby inhibiting normal bacterial proliferation. Furthermore, MEO’s antioxidant properties were evaluated through its scavenging effects on DPPH and hydroxyl radicals, as well as its ferric reducing antioxidant power (FRAP). The findings revealed that MEO exhibited the strongest scavenging activity against DPPH radicals, followed by hydroxyl radical scavenging, with the FRAP results being comparatively weaker. These results suggest that MEO not only possesses potent antibacterial effects but also exhibits notable antioxidant activity, indicating potential for broader applications.

## 1 Introduction

For a long time, natural plant essential oils have been recognized for their wide-ranging biological activities, including antibacterial, antifungal, and antioxidant properties. Essential oils are complex mixtures of volatile compounds, primarily com-posed of terpenoids, phenols, and other bioactive constituents (Wu et al., 2020; Hadidi et al., 2020; Bouyahya et al., 2017). These compounds exhibit various therapeutic characteristics, making essential oils an attractive focus for researchers seeking alternative and effective treatments, particularly against drug-resistant pathogens (Der Torossian Torres and de la Fuente-Nunez, 2019). Rich in terpenoids, these essential oils demonstrate potent antibacterial effects by disrupting the integrity of microbial cell walls and membranes, leading to cell lysis and ultimately resulting in bacterial death (Visan and Negut, 2024).

Magnolia essential oil (MEO), extracted from the dried flower buds of *Magnolia biondii Pamp.*, *Magnolia denudata Desr.*, and *Magnolia sprengeri Pamp.*, is a volatile oil rich in bioactive compounds such as eugenol, α-pinene, and linalool. Traditionally, these flower buds have been used in Chinese medicine to dispel wind-cold, alleviate nasal congestion, and relieve headaches caused by cold exposure. Beyond its conventional medicinal uses, recent studies have demonstrated that MEO exhibits significant antibacterial, anti-inflammatory, and antioxidant properties, making it a promising natural agent for various applications. In the field of food preservation, MEO has been recognized for its ability to inhibit foodborne pathogens and delay spoilage by targeting bacterial cell membranes and oxidative stress pathways. Its antimicrobial efficacy, coupled with its antioxidant capacity, suggests potential for extending the shelf life of perishable foods while maintaining their quality and safety, and its volatile nature allows for application in vapor-phase antimicrobial packaging systems, offering an alternative to synthetic preservatives (Liu Y. et al., 2024). Meanwhile, in the pharmaceutical industry, MEO’s anti-inflammatory and immunomodulatory effects have drawn attention for treating respiratory conditions, including rhinitis and allergic reactions. While some studies have explored its role in traditional medicine, research into its precise antibacterial mechanisms and interactions with bacterial metabolism remains insufficient. A deeper understanding of these mechanisms could pave the way for novel antimicrobial agents derived from MEO, addressing the growing concern of antibiotic resistance. By expanding the exploration of MEO beyond traditional applications, this study aims to further elucidate its potential in both food preservation and medicinal fields, contributing to the broader utilization of natural plant-based antimicrobials.

Bacteria such as *Escherichia coli* (*E. coli*), *Staphylococcus aureus* (*S. aureus*), *Listeria monocytogenes* (*L. monocytogenes*), and *Salmonella typhimurium* (*S. typhimurium*) are common foodborne pathogens that can cause severe health issues when contaminating food (Oh et al., 2017). These bacteria are capable of inducing symptoms ranging from food poisoning and gastroenteritis to fever, posing significant risks, especially to vulnerable populations like the elderly, pregnant women, and children. In recent decades, the misuse and overuse of antibiotics have led to a rapid rise in antibiotic resistance, with the World Health Organization recognizing antimicrobial resistance as one of the top global public health threats (King et al., 2019). Multidrug-resistant strains of these pathogens have emerged, rendering conventional treatments increasingly ineffective and leading to higher morbidity, mortality, and economic burden worldwide. As antibiotic resistance becomes a more pressing concern, there is an urgent need to develop safe, natural antibacterial agents that are less likely to induce resistance. Essential oils derived from plants, known for their broad-spectrum antimicrobial properties, have emerged as promising alternatives (Sánchez-Hidalgo et al., 2018; Khaliullina et al., 2024). These natural substances can effectively disrupt bacterial cell structures and inhibit metabolic pathways, offering a potential solution to the growing problem of antibiotic resistance.

This study identified terpenoids as the major components of MEO via GC-MS analysis and revealed its antibacterial mechanism, which primarily involves disrupting bacterial cell membranes, leading to metabolic dysfunction, lipid peroxidation, and oxidative stress, ultimately inhibiting bacterial growth and reproduction, while in vitro assays also confirmed its antioxidant activity. By integrating enzyme activity assays and membrane integrity tests, this study provides a more comprehensive molecular-level understanding of MEO’s antibacterial effects compared to previous research, which mainly focused on its chemical composition and therapeutic applications in rhinitis. Given the rising concern over antibiotic resistance, these findings highlight MEO’s potential as a natural antibacterial agent with a lower risk of resistance development, making it a promising alternative to traditional antibiotics.

## 2 Materials and methods

### 2.1 Materials and reagents

Magnolia essential oil was obtained from Jiangxi Cedar Natural Medicinal Oil Co., Ltd. in Jiangxi, China. Nutrient broth (NB) and nutrient agar (NA) purchased from Guangdong Huankai Biotechnology Co., Ltd (Guangzhou, China). *Escherichia coli* (GDMCC NO.1.1917), *Staphylococcus aureus* (GDMCC NO. 1.221), *Listeria monocytogenes* (GDMCC NO. 1.2408) and *Salmonella typhimurium* (GDMCC NO. 1.237) were obtained from Guang-dong Microbial Culture Collection Center (Guangzhou, China). Alkaline Phosphatase (AKP), 1,1-Diphenyl-2-Picrylhydrazyl Radical (DPPH), hydroxyl radicals, Ferric Ion Reducing Antioxidant Power (FRAP) and ATPase (ATP) kits were purchased from Nanjing Jiancheng Bioengineering Institute (Nanjing, China). Malondialdehyde (MDA) and superoxide dismutase (SOD) kits were purchased from Solarbio science & technology Co., Ltd. (Beijing, China). Other chemical reagents were of analytical grade and purchased from Sinopharm Chemical Reagent Co., Ltd. (Shanghai, China).

### 2.2 Chemical composition analysis of MEO

The GC-MS analysis was conducted utilizing an Agilent 6890N-5973 gas chromatography mass spectrometer equipped with an HP-INNOWax model gas chromatography column (Li et al., 2023). In a 20 mL extraction bottle, 1 g of MEO was sealed and immersed in a 60°C water bath with magnetic stir-ring set at 500 rpm. After a 20 min equilibration period, extraction was carried out for an additional 30 min upon insertion of the extraction needle. Prior to use, the extraction needle was activated at the gas injection port for 20 min at 250°C. The temperature program included an inlet temperature of 250°C, GC interface temperature of 250°C, carrier gas flow rate of 1.5 mL/min, and a split ratio of 4:1. The temperature program was as follows: initially set at 40°C, held for 5 min, ramped at 5°C/min to 250°C, and then held for 10 min. MS conditions comprised an ion source temperature of 230°C, quadrupole temperature of 150°C, EI ionization at 70 eV, and a full scan from 35–550 m/z. Identification of compounds within MEO was accomplished using the National Institute of Standards and Technology (NIST14) database alongside literature references. The relative content of each compound in the chromatogram was determined employing the area normalization method.

### 2.3 Antibacterial activity

#### 2.3.1 Bacterial liquid incubate

The frozen strains of *E. coli*, *S. aureus*, *L. monocytogenes* and *S. typhimurium* were revived by inoculating them on NA solid medium. Single colonies were picked and transferred into NB liquid medium, where they were cultured at 37°C for 24 h (Su et al., 2023). The bacterial cultures were then stored at 4°C. Before starting the experiments, the bacterial suspensions were adjusted to a concentration of 10^8^ CFU/mL using a McFar-land turbidity standard.

#### 2.3.2 Method for assessing antimicrobial activity

The antibacterial activity of MEO against common foodborne bacteria (*E. coli*, *S. aureus*, *L. monocytogenes*, and *S. typhimurium*) was evaluated using the filter paper disk diffusion method (El Barnossi et al., 2020). A 100 μL bacterial suspension with a concentration of 1 × 10^6^ CFU/mL was spread evenly on NA agar plates using a sterile spreader. Sterile filter paper disks were gently placed at the center of each plate. MEO (5 μL) was added to the filter paper disks, and sterile water was used as a blank control. The plates were incubated at 37°C for 24 h. The inhibition zones were measured and photographed. This procedure was repeated three times for accuracy.

#### 2.3.3 The MIC and MBC of MEO to bacteria

The MIC and MBC of MEO were determined using the microdilution method (Veiga et al., 2019). Initially, a solution of MEO in NB medium was prepared at a concentration of 60 μL/mL, with 4% DMSO added as a cosolvent. This solution was then further diluted to concentrations of 10, 9, 8, 7, 6, 5, 4, 3, 2 and 1 μL/mL. In a sterile 96-well plate, 100 μL of the diluted MEO solution was added to each well from left to right, followed by the addition of 100 μL of bacterial suspension (1 × 10^6^ CFU/mL), gently mixing the contents. The plate was then incubated at 37°C for 24 h in a constant-temperature microbiological incubator. The bacterial growth in each well was observed and analyzed, with the lowest concentration showing no bacterial growth and no increase in optical density (OD) defined as the MIC value (Kowalska-Krochmal and Dudek-Wicher, 2021). The incubation continued for an additional 48 h, and the growth was reassessed. The lowest concentration with no bacterial growth and no increase in OD was defined as the MBC value. The experiment was repeated three times. The blank group did not contain MEO or DMSO, while the control group only included DMSO.

#### 2.3.4 Growth curve

The determination of growth curve referred to the method of Feng et al. (2022). The effect of MEO on the growth curve of the test bacteria was assessed in a sterile 96-well plate. MEO solutions at concentrations of 0.25 MIC, 0.5 MIC, and 1 MIC, determined from preliminary experiments, were added sequentially, with 200 μL of each solution introduced into the wells. NB medium without MEO served as the blank control. Subsequently, 20 μL of bacterial suspension (1 × 10^6^ CFU/mL) was added to each well, and the plate was incubated at 37°C in a shaking water bath at 100 rpm. The OD at 600 nm was measured and recorded every 2 h to plot the growth curve. Each group was tested in triplicate, and the bacterial growth curve was represented by the average turbidity, with absorbance corresponding to the time intervals.

#### 2.3.5 Nucleic acid and protein leakage assay

The determination of the contents is based on the method of Liu et al. (2020). In a 10 mL suspension of test bacteria (1 × 10^6^ CFU/mL), MEO was added, using 4% DMSO as a co-solvent, to achieve a final concentration equal to the MIC. The blank group consisted of samples without MEO or DMSO, while the control group included only DMSO. The bacterial suspension was then incubated at 37°C in a shaking water bath at 100 rpm for 2 h. Following incubation, the suspension was centrifuged at 8000 rpm for 10 min to collect the supernatant. This supernatant was transferred to fresh tubes for analysis, where the optical density at 260 and 280 nm was measured to quantify the nucleic acids and proteins released from the cytoplasm.

#### 2.3.6 Electric conductivity assay

The solution conductivity was determined according to the method of Wingfield (2014). In a 10 mL suspension of test bacteria (1 × 10^6^ CFU/mL), MEO was added, with 4% DMSO used as a co-solvent, to achieve a final concentration corresponding to the MIC. The blank group consisted of samples without MEO or DMSO, while the control group included DMSO only. The bacterial suspension was incubated at 37°C in a shaking water bath at 100 rpm for 2 h. After incubation, the suspension was centrifuged at 8,000 rpm for 10 min to collect the supernatant, which was then transferred to fresh tubes. The conductivity of each tube’s supernatant was measured using a conductivity meter.

#### 2.3.7 Biochemical index assay

The determination of intracellular enzymes referred to previous research methods (Shi et al., 2016; Rubeena et al., 2020; Li et al., 2019). In a 10 mL suspension of test bacteria (1 × 10^6^ CFU/mL), MEO was added, along with 4% DMSO as a co-solvent, to achieve a final concentration at the MIC. The blank group consisted of samples without MEO or DMSO, while the control group included DMSO only. The bacterial suspension was then incubated at 37°C in a shaking water bath at 100 rpm for 2 h. Following this, the suspension was centrifuged at 8,000 rpm for 10 min, discarding the supernatant and collecting the bacterial pellet. High-efficiency RIPA lysis buffer was added to the cells, and a cell disruptor was used to thoroughly lyse the cells while in an ice bath. The lysate was centrifuged again at 8,000 rpm for 10 min, and the supernatant was transferred to a new tube and kept on ice for subsequent biochemical assays. The detection steps for AKP, ATP, MDA, and SOD activities are detailed in the kit instructions.

#### 2.3.8 Scanning electron microscope (SEM) assay

To confirm and validate the results observed under an optical microscope, we slightly modified the previously reported method of observing test bacteria using SEM (Bai et al., 2019). The bacteria (at a concentration of 10^8^ CFU/mL) were treated with MEO at MIC concentration for 2 h at 37°C. The precipitate was then centrifuged at 6,000 rpm for 10 min and washed three times with PBS. The collected cells were fixed in 2.5% glutaraldehyde at 4°C for 24 h, followed by dehydration using a series of ethanol concentrations (15%, 30%, 45%, 60%, 75%, 90%, and 100%) for 10 min each. Finally, the dehydrated samples were gold-coated and examined using SEM. A control experiment was conducted without MEO treatment.

### 2.4 Antioxidant activity

#### 2.4.1 1,1-diphenyl-2-picrylhydrazyl (DPPH) radical scavenging activity assay

To prepare the MEO solutions, anhydrous ethanol was used to create concentration gradients of 1.25, 2.5, 5, 10, 15, 20, and 40 mg/mL. A 5 mg/mL BHT solution served as the positive control. For each test, 0.5 mL of the sample solution was mixed with an equal volume of a 0.2 mM DPPH anhydrous ethanol solution. The mixture was thoroughly mixed and then placed in the dark for 30 min, with three parallel operations for each concentration. The absorbance was measured at a wavelength of 517 nm (Ashmawy et al., 2024). The experiment was repeated three times. The DPPH radical scavenging rate was calculated using the following formula (1):


$$\mathrm{DPPH}\,\mathrm{radical}\,\mathrm{scavenging}\,\mathrm{activity}\,\left[1-\frac{\left(A_{1}-A_{0}\right)}{A_{2}}\right]\,100\%$$

A_0_ represents the absorbance of the sample group (sample without DPPH solution), A_1_ represents the absorbance of the sample group containing DPPH, and A_2_ rep-resents the absorbance of the control group (sample without DPPH solution).

#### 2.4.2 Hydroxyl radical scavenging activity assay

To prepare the MEO solutions, anhydrous ethanol was used to create concentration gradients of 1.25, 2.5, 5, 10, 15, 20, and 40 mg/mL, with a 5 mg/mL BHT solution serving as the positive control. A 9.0 mmol/L FeSO4 solution, a 9.0 mmol/L ethanol-salicylic acid solution, and a 3% H2O2 solution were prepared and stored after thorough mixing. In a test tube, 1 mL of the sample solution was taken, followed by the addition of 1 mL of the prepared FeSO4 solution and 1 mL of the ethanol-salicylic acid solution. Finally, 1 mL of the H2O2 solution was added. The mixture was shaken thoroughly and incubated in a water bath at 37°C for 30 min. The absorbance was then measured at a wavelength of 510 nm (Zhu et al., 2024). This experiment was repeated three times. The hydroxyl radical scavenging rate was calculated using the following formula (2):


$$\mathrm{Hydroxyi}\,\mathrm{radical}\,\mathrm{scavenging}\,\mathrm{activity}\,\left[1-\frac{\left(A_{1}-A_{2}\right)}{A_{0}}\right]\,100\%$$

A_0_ represents the absorbance value of distilled water participating in the reaction instead of the sample solution. A_1_ represents the absorbance value of the sample solution participating in the reaction. A_2_ represents the absorbance value of the sample solution and distilled water instead of 3% H2O2 in the reaction.

#### 2.4.3 Ferric ion reducing antioxidant power (FRAP) assay

To prepare the MEO solutions, anhydrous ethanol was used to create concentration gradients of 1.25, 2.5, 5, 10, 15, 20, and 40 mg/mL, with a 5 mg/mL BHT solution serving as the positive control. In a 10 mL centrifuge tube, 1 mL of the solution was centrifuged, and then 2.5 mL of phosphate buffer (0.2 mol/L, pH 6.6) and 2.5 mL of 1% potassium ferricyanide solution were added. The mixture was thoroughly mixed and reacted at 50°C for 20 min before being rapidly cooled. Next, 2.5 mL of 10% tri-chloroacetic acid solution was added, followed by centrifugation at 4,000 rpm for 15 min. The supernatant (2.5 mL) was then taken and mixed with 2.5 mL of distilled water and 0.5 mL of 0.1% ferric chloride solution. After sufficient mixing, the reaction was allowed to proceed for 10 min, and the absorbance was measured at a wave-length of 700 nm. This experiment was repeated three times to ensure accuracy and reliability (Chebbac et al., 2023).

### 2.5 Statistical analysis

All experimental procedures were repeated three times. All data are presented as mean ± standard deviation (SD). Graphs were created using GraphPad Prism 10, and statistical analysis including one-way analysis of variance (ANOVA) and Duncan’s multiple range test was conducted using SPSS 27 software. *P* < 0.05 was considered as statistically significant.

## 3 Results

### 3.1 Chemical component analysis of MEO

The chemical composition of essential oil was analyzed using GC-MS, with the results presented in Table 1. A total of 49 compounds were identified from the database search, accounting for 99.04% of the total essential oil content. The major components with higher concentrations include 1,8-cineole (44.87%), (+)-citronellal (6.93%), and linalool (29.1%). The primary constituents of MEO are terpenoids.

**TABLE 1:** The chemical component analysis of magnolia essential (MEO).

ID	RT (min)	Compound name	CAS no.	Relative content (%)
1	8.827	α-Pinene	80-56-8	0.25
2	10.107	Camphene	79-92-5	0.07
3	11.600	β-Pinene	127-91-3	0.30
4	11.935	α-Phellandrene	99-83-2	0.17
5	12.082	β-Phellandrene	555-10-2	0.47
6	13.603	Sabinene	3387-41-5	0.81
7	13.997	α-Terpipene	99-86-5	3.72
8	14.555	Limonene	138-86-3	2.97
9	15.889	1,8-cineole	470-82-6	44.87
10	16.283	γ-Terpinene	99-85-4	0.04
11	16.459	β-(Z)-ocimene	3338-55-4	0.04
12	17.005	p-Cymene	99-87-6	0.91
13	17.323	α-Terpinolene	586-62-9	0.03
14	17.611	Octanal	124-13-0	0.01
15	18.944	6-Methyl-5-hepten-2-one	110-93-0	0.03
16	19.414	2,6-Dimethyl-5-heptenal	106-72-9	0.03
17	21.870	Cis-Linalool oxide	1365-19-1	0.02
18	22.352	3-Furaldehyde	498-60-2	0.02
19	22.570	Trans-Linalool oxide	34995-77-2	0.01
20	23.116	(+)-Citronellal	2385-77-5	6.93
21	25.031	Linalool	78-70-6	29.10
22	25.566	Isopulegol	89-79-2	0.43
23	25.930	β-Elemene	515-13-9	0.73
24	26.048	Cubebene	13744-15-5	0.06
25	26.142	Trans-Caryophyllene	87-44-5	0.03
26	26.324	Citronellyl formate	105-85-1	0.01
27	26.788	(+)-Epi-bicyclosesquiphellandrene	54324-03-7	0.02
28	27.317	Citronellyl acetate	150-84-5	0.55
29	27.640	α-Humulene	6753-98-6	0.08
30	27.910	Z-Citral	106-26-3	0.04
31	27.999	γ-Muurolene	30021-74-0	0.09
32	28.134	α-Terpineol	98-55-5	0.04
33	28.222	Geranyl formate	105-86-2	0.02
34	28.586	Germacrene-d	23986-74-5	0.44
35	28.792	α-Muurolene	10208-80-7	0.23
36	29.015	Geranial	141-27-5	0.08
37	29.415	Geranyl acetate	105-87-3	0.41
38	29.638	Citronellol	106-22-9	2.80
39	30.296	Nerol	106-25-2	0.03
40	30.372	α-Cadinene	24406-05-1	0.04
41	31.177	Calamenene	483-77-2	0.02
42	31.412	Geraniol	106-24-1	2.44
43	31.665	Geranylacetone	3796-70-1	0.02
44	33.357	(E)-2,6-Dimethylocta-3,7-diene-2,6-diol	51276-34-7	0.03
45	36.166	Elemol	639-99-6	0.20
46	37.746	Eugenol	97-53-0	0.20
47	37.858	γ-Eudesmol	1209-71-8	0.08
48	38.181	T-Muurolol	19912-62-0	0.02
49	39.021	α-Cadinol	481-34-5	0.03
	Total	–	–	99.04

**FIGURE 1:**
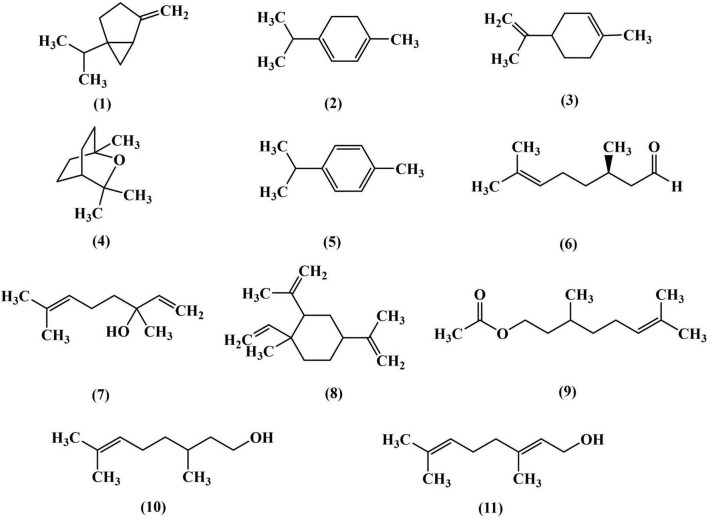
Structural formula of chemical components with content greater than 0.5% in magnolia essential oil (MEO).

Based on the chemical composition obtained in Table 1 and the database search results, Figure 1 illustrates the structural formulas of monomeric compounds with a relative content greater than 0.5%.

### 3.2 Antibacterial activity assay

#### 3.2.1 Determination of inhibition zone

**FIGURE 2:**
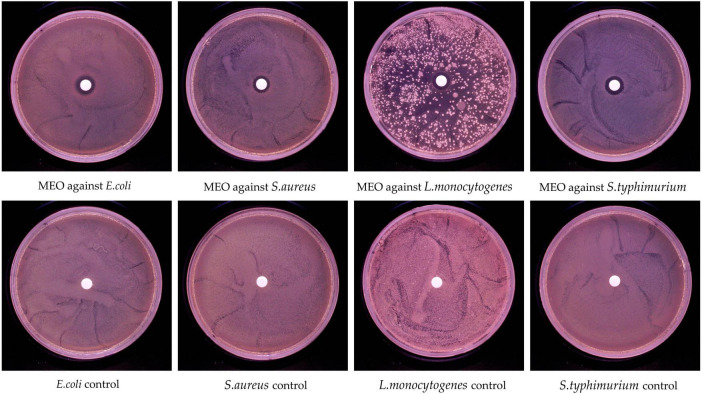
*In vitro* antibacterial activity of magnolia essential oil (MEO).

The inhibition ability of MEO was evaluated using the disk diffusion method. As shown in Figure 2, MEO exhibited growth inhibition against *E. coli*, *S. aureus*, *L. monocytogenes*, and *S. typhimurium*. The diameters of the inhibition zones are presented in Table 2, with the inhibition zones for *E. coli*, *S. aureus*, *L. monocytogenes*, and *S. typhimurium* measured at 12.63 ± 0.35 mm, 10.94 ± 0.44 mm, 12.63 ± 0.35 mm, and 12.5 ± 0.24 mm, respectively. These results demonstrate that MEO has significant antibacterial potential.

**TABLE 2:** Inhibition zone determination of magnolia essential oil (MEO) against four tested bacterial strains.

Group	Diameter of bacterial inhibition zone of different system categories (mm)			
	***E. coli***	***S. aureus***	***L. monocytogenes***	***S. typhimurium***
Control	-	-	-	-
MEO	12.63 ± 0.35^a^	10.94 ± 0.44^b^	11.34 ± 0.53^b^	12.5 ± 0.24^a^

“-” It means no bacteriostatic effect. Different letters in the same figure indicate statistically significant difference at *P* < 0.05 between different samples. Each value represents the mean of three replicates ± standard deviation.

#### 3.2.2 The MIC and MBC of MEO to bacteria

The MIC and MBC values of MEO against four bacterial strains were determined using the broth microdilution method, with the results shown in Table 3. The MIC values for *E. coli*, *S. aureus*, *L. monocytogenes*, and *S. typhimurium* were 5, 5, 4, and 4 μL/mL, respectively. The corresponding MBC values were 5, 8, 5, and 5 μL/mL, respectively. These results indicate that MEO exhibits relatively weaker bactericidal activity against *S. aureus* compared to the other strains. In the control group, bacterial growth was normal, confirming that the addition of a certain amount of DMSO had no inhibitory effect on bacterial growth.

**TABLE 3:** The minimum inhibitory concentration (MIC) and minimum bactericidal concentration (MBC) of magnolia essential oil (MEO) against four tested bacterial strains.

Bacteria	MIC (μL/mL)			MBC (μL/mL)
	**Blank**	**Control**	**Test**	
*E. coli*	-	-	5	5
*S. aureus*	-	-	5	8
*L. monocytogenes*	-	-	4	5
*S. typhimurium*	-	-	4	5

“-” It means no bacteriostatic effect.

#### 3.2.3 Growth curve

**FIGURE 3:**
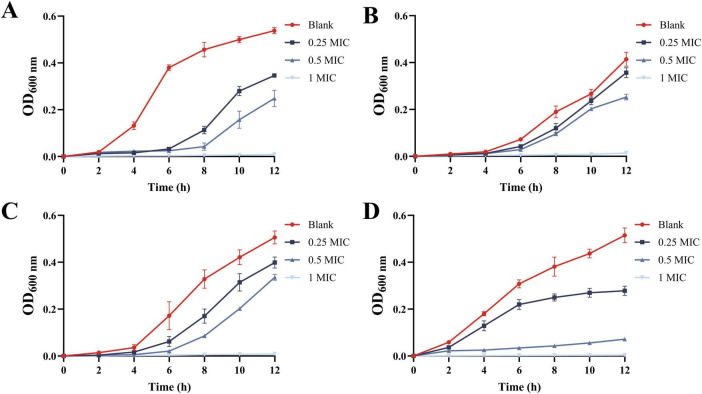
The growth curves of *E. coli* **(A)**, *S. aureus* **(B)**, *L. monocytogenes* **(C)**, and *S. typhimurium* **(D)** were treated with different concentrations of magnolia essential oil (MEO). Each value represents the mean of three replicates ± standard deviation.

The effect of MEO on the growth of *E. coli*, *S. aureus*, *L. monocytogenes*, and *S. typhimurium* is shown in Figure 3. In the control group, all four bacteria rapidly entered the logarithmic growth phase. However, treatment with 0.25 MIC and 0.5 MIC of MEO effectively delayed the logarithmic growth phase for all test bacteria. At the 12 h mark, different concentrations of MEO exhibited inhibitory effects on the four bacterial strains. The optical density (OD) values at 1 MIC remained almost unchanged within 12 h, indicating that the test bacteria were highly sensitive to this concentration. Significant differences were observed between the MEO-treated groups and the control group.

#### 3.2.4 Nucleic acid and protein leakage

**FIGURE 4:**
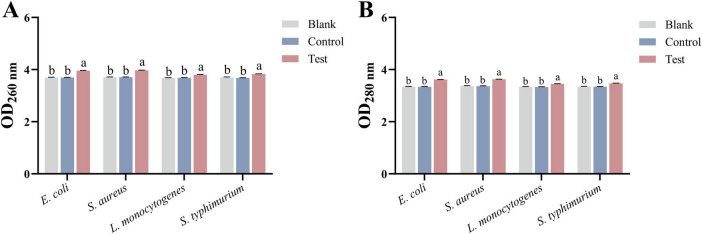
**(A)** Is the nucleic acid released by *E. coli*, *S. aureus*, *L. monocytogenes*, and *S. typhimurium* after treatment with different groups. **(B)** Is the protein released by *E. coli*, *S. aureus*, *L. monocytogenes*, and *S. typhimurium* after treatment with different groups. Different letters in the same figure indicate statistically significant difference at *P* < 0.05 between different samples. Each value represents the mean of three replicates ± standard deviation.

The release of intracellular substances after MEO treatment was measured using UV spectrophotometry. As shown in Figure 4, after incubating the four bacterial strains with MEO for 2 h, the absorbance at 260 and 280 nm significantly increased. A statistically significant difference (*p* < 0.05) was observed between the test group and both the blank and control groups. The rise in extracellular nucleic acids and proteins suggests that MEO disrupts the permeability of the bacterial cell membranes, leading to the leakage of intracellular materials and consequently inhibiting bacterial growth and reproduction.

#### 3.2.5 Electric conductivity

**FIGURE 5:**
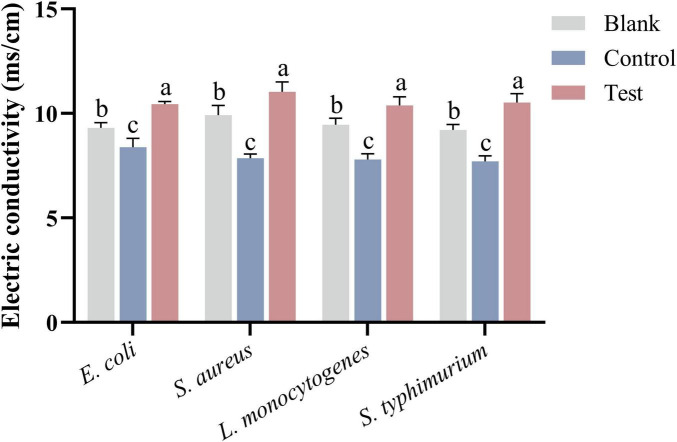
The electric conductivity of bacterial culture medium after treatment of *E. coli*, *S. aureus*, *L. monocytogenes*, and *S. typhimurium* in different groups. Different letters in the same figure indicate statistically significant difference at *P* < 0.05 between different samples. Each value represents the mean of three replicates ± standard deviation.

The electric conductivity of bacterial cultures was measured after incubation with MEO using a conductivity meter. As shown in Figure 5, after 2 h of incubation, the conductivity of the test group significantly increased compared to the blank group (*p* < 0.05), which aligns with the findings in Figure 4. This increase in conductivity is due to the leakage of intracellular substances such as nucleic acids and proteins, which carry charges and elevate the ionic concentration in the culture medium. In contrast, the conductivity of the control group slightly decreased compared to the blank group, likely because DMSO, a non-ionic solvent, does not contribute to ion concentration and may reduce the number of conductive ions per unit volume.

#### 3.2.6 AKP activity

**FIGURE 6:**
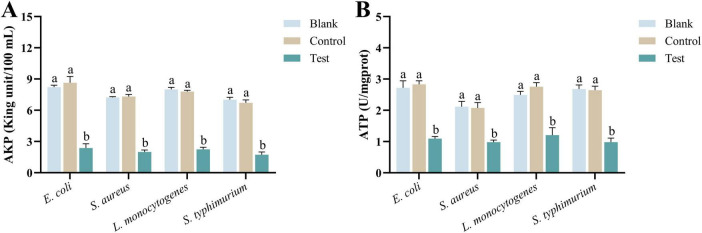
**(A)** Is the alkaline phosphatase (AKP) enzyme activity of *E. coli*, *S. aureus*, *L. monocytogenes*, and *S. typhimurium* in different groups. **(B)** Is the ATPase activity of *E. coli*, *S. aureus*, *L. monocytogenes*, and *S. typhimurium* in different groups. Different letters in the same figure indicate statistically significant difference at *P* < 0.05 between different samples. Each value represents the mean of three replicates ± standard deviation.

Alkaline phosphatase enzyme is closely related to bacterial metabolic activity, membrane stability, and the synthesis and degradation of the cell wall. When the level of AKP decreases, it indicates damage to the bacterial cell membrane or wall, leading to reduced metabolic activity and slower growth. Therefore, a reduction in AKP enzyme levels can be considered a marker of bacterial damage (Zhou et al., 2022; Shi et al., 2024). As shown in Figure 6A, after 2 h of co-incubation with MEO, the AKP levels in all four test bacteria were significantly reduced. Furthermore, there was a notable difference between the test group and the blank and control groups (*p* < 0.05). This suggests that MEO has a destructive effect on the bacterial cell wall.

#### 3.2.7 ATP activity

ATPase plays a crucial role in bacterial cells by hydrolyzing ATP into ADP, releasing energy necessary for various cellular processes. When the level of ATPase decreases, the bacteria experience multifaceted damage (Mao et al., 2024). As illustrated in Figure 6B, after 2 h of incubation with MEO, the ATPase content in all four test bacteria showed a significant reduction. Moreover, there was a noticeable difference between the experimental group and the blank and control groups (*p* = 0.05). A reduction in ATPase levels leads to insufficient energy metabolism, impaired transmembrane ion transport, membrane potential imbalance, and weakened stress responses. In severe cases, these disruptions can inhibit bacterial growth or even lead to cell death.

#### 3.2.8 MDA activity

**FIGURE 7:**
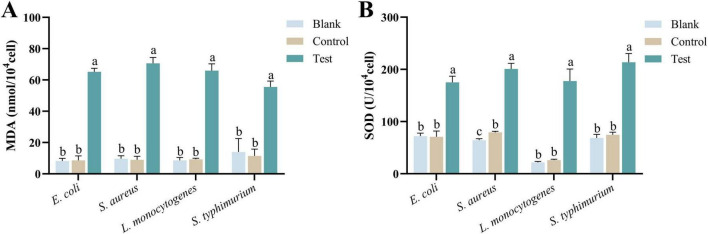
**(A)** Is the malondialdehyde (MDA) content of different groups of *E. coli*, *S. aureus*, *L. monocytogenes*, and *S. typhimurium*. **(B)** Is the superoxide dismutase (SOD) activity of different groups of *E. coli*, *S. aureus*, *L. monocytogenes*, and *S. typhimurium*. Different letters in the same figure indicate statistically significant difference at *P* < 0.05 between different samples. Each value represents the mean of three replicates ± standard deviation.

Malondialdehyde is one of the primary markers of lipid peroxidation in cell membranes, typically produced under oxidative stress conditions. An increase in MDA levels often indicates that the bacteria have undergone oxidative damage or that their membrane structure has been compromised (Suo et al., 2022; Chen et al., 2022). As shown in Figure 7A, after 2 h of co-incubation with MEO, the MDA content in the four test bacteria significantly increased. There was a marked difference between the experimental group and the blank and control groups (*p* < 0.05). The increase in MDA levels is a sign of severe oxidative damage to bacterial cells, representing structural damage to the membrane and heightened oxidative stress, which negatively impacts bacterial growth and reproduction.

#### 3.2.9 SOD activity

Superoxide dismutase primarily functions to eliminate superoxide anions in bacteria by catalyzing their conversion into hydrogen peroxide and oxygen, thereby protecting cells from oxidative damage. An increase in SOD levels typically indicates that the bacteria are experiencing oxidative stress (Mohammad et al., 2014). As shown in Figure 7B, after 2 h of co-incubation with MEO, the SOD enzyme content significantly increased in the four test bacteria. There were statistically significant differences between the experimental group and the blank and control groups (*p* < 0.05). The elevated SOD levels reflect the bacterial response to oxidative stress, indicating that the bacteria are under environmental pressure, which may involve membrane damage, oxidative damage to proteins and DNA, and metabolic dysfunction.

#### 3.2.10 Scanning electron microscope

**FIGURE 8:**
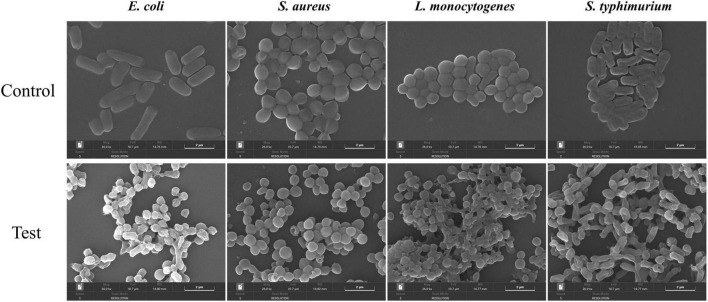
The effects of different groups of *E. coli*, *S. aureus*, *L. monocytogenes*, and *S. typhimurium* on bacterial morphology.

The impact of MEO on the cellular morphology of the four test bacteria was observed using SEM. As shown in Figure 8, the control group displayed bacteria with smooth, intact surfaces, regular edges, full bodies, and tightly packed structures. However, after treatment with MEO at the MIC level, varying degrees of deformation were evident in all four bacteria. The bacterial surfaces became unclear, collapsed, with cell membrane indentations, ruptures, and distorted shapes. These results indicate that MEO damaged the bacterial cell wall and membrane structures. The disruption of these structures led to the leakage of intracellular contents, compromising bacterial integrity and resulting in its antibacterial effect.

### 3.3 Antioxidant activity

**FIGURE 9:**
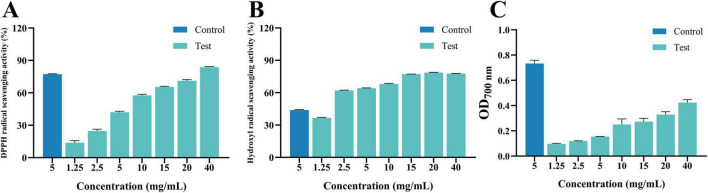
**(A)** Is 1,1-diphenyl-2-picrylhydrazyl radical (DPPH) radical scavenging activity of magnolia essential oil (MEO). **(B)** Is hydroxyl radical scavenging activity of MEO. **(C)** Is Ferric Ion Reducing Antioxidant Power activity of MEO. Each value represents the mean of three replicates ± standard deviation.

As shown in Figure 9, within the tested concentration range, the radical scavenging activity of MEO against DPPH, hydroxyl radicals, and FRAP exhibited a dose-dependent relationship. The scavenging rate increased with increasing MEO concentration, indicating that its effect was concentration-dependent. Among the measured antioxidant capacities, MEO demonstrated the highest scavenging activity against DPPH (IC_50_ = 7.421 mg/mL), followed by hydroxyl radicals (IC_50_ = 1.794 mg/mL), while FRAP exhibited the lowest activity. This suggests that MEO possesses a stronger ability to neutralize DPPH radicals compared to its reducing power as measured by hydroxyl radical and FRAP assays.

## 4 Discussion

Plant-derived natural medicines have long been valued for their diverse biological activities, including antibacterial, antioxidant, anti-inflammatory, and antiviral properties (Zhang et al., 2024). Compared with traditional antibiotics, essential oils (EOs) are gaining attention due to their broad-spectrum antimicrobial efficacy and lower risk of inducing bacterial resistance (Wells, 2024). Previous studies have demonstrated that terpenoids and phenolic compounds in EOs can disrupt bacterial membranes, alter ion gradients, and interfere with key metabolic pathways, ultimately leading to cell death (Visan and Negut, 2024). Among them, tea tree oil (Melaleuca alternifolia) and lavender oil (Lavandula angustifolia) are well-known for their strong antibacterial and antioxidant properties, with proven effectiveness against foodborne pathogens (Kokina et al., 2019). The present study confirms that MEO exhibits similar antibacterial activity against *Escherichia coli*, *Staphylococcus aureus*, *Listeria monocytogenes*, and *Salmonella typhimurium*, with its primary active components—1,8-cineole, citronellal, and linalool—playing a crucial role (György et al., 2020). Compared with other plant EOs, MEO demonstrated notable antibacterial potential against both Gram-positive and Gram-negative bacteria, suggesting that its bioactive compounds may have broader applications in food safety and medical fields.

The increasing prevalence of antibiotic-resistant bacteria has become a major global health concern, particularly in foodborne pathogens such as *E. coli*, *S. aureus*, and *L. monocytogenes* (Bajrami et al., 2024). This has driven the search for alternative antimicrobial agents that are both effective and safe. In this study, inhibition zone determination, MIC, and MBC assays confirmed the potent antibacterial effects of MEO, with results comparable to or even exceeding those of previously studied EOs. Growth curve analysis further demonstrated that MEO significantly prolonged the logarithmic growth phase of bacteria, indicating a bacteriostatic effect. Moreover, MEO was found to cause substantial leakage of intracellular materials, induce lipid peroxidation, and generate oxidative stress, ultimately impairing bacterial metabolism and survival. These antibacterial mechanisms align with those reported for tea tree and cinnamon oils (Graziano et al., 2016), reinforcing the hypothesis that essential oils primarily act by disrupting bacterial membrane integrity. In addition to its antibacterial activity, MEO exhibited antioxidant properties, as evidenced by its strong scavenging effect on DPPH and hydroxyl radicals, although its ferric reducing antioxidant power (FRAP) was relatively weaker. While the antioxidant activities of tea tree and lavender oils have been widely studied, further research is needed to fully understand the antioxidative mechanisms of MEO and its potential applications in preventing oxidative deterioration in food systems.

Despite its promising antimicrobial and antioxidant properties, several challenges must be addressed before MEO can be widely applied in food preservation and healthcare. One major concern is its stability, as essential oils are highly volatile and susceptible to degradation under environmental factors such as heat, light, and oxygen exposure (Liu L. et al., 2024). Additionally, the potential cytotoxicity of MEO at high concentrations should be thoroughly evaluated to ensure its safety for human consumption. Furthermore, while essential oils have been explored as natural food preservatives, their strong aroma and hydrophobic nature may limit direct application in food products (Shaukat et al., 2023). To overcome these limitations, future research should focus on enhancing the stability and controlled release of MEO through nanoencapsulation or emulsification techniques. Additionally, investigating the synergistic effects of MEO with other natural antimicrobials, such as organic acids or plant polyphenols, may help improve its efficacy while reducing the required concentration (Timbe et al., 2021). Lastly, comprehensive toxicological studies and clinical evaluations are necessary to confirm its long-term safety and effectiveness. By addressing these challenges, MEO has the potential to become a viable natural alternative to synthetic preservatives and antibiotics, contributing to safer and more sustainable solutions in food preservation and medical applications.

## 5 Conclusion

In this study, the chemical constituents of MEO were analyzed by GC-MS. The highest proportion of compounds was terpenoids, and the main components with higher content were 1,8-cineole (44.87%), (+) -Citronellal (6.93%) and Linalool (29.1%). Through the determination of inhibition zone, MIC and MBC, it was preliminarily concluded that MEO had good antibacterial effect on the four test bacteria. The growth curve shows that MEO has an inhibitory effect on the growth and reproduction of the test bacteria, and can effectively delay the logarithmic growth period of the bacteria. Through the preliminary exploration of the bacterial mechanism, it was found that MEO mainly acts on the initial stage of bacterial growth. By destroying the cell membrane, they cause a large amount of material loss in bacteria, affect bacterial metabolic activity, cause peroxidation of bacteria, stimulate their oxidative stress, and cannot maintain the normal growth and reproduction of bacteria. The scavenging ability of MEO on DPPH and hydroxyl radicals and FRAP were also determined. MEO had the best scavenging effect on DPPH, followed by hydroxyl radical scavenging effect, and FRAP had the worst effect.

## Data Availability

The raw data supporting the conclusions of this article will be made available by the authors, without undue reservation.
